# Pan-cancer analysis and experimental validation identify ndc1 as a potential immunological, prognostic and therapeutic biomarker in pancreatic cancer

**DOI:** 10.18632/aging.205048

**Published:** 2023-09-20

**Authors:** Qian Shen, Junchen Li, Chuanlong Zhang, Xue Pan, Yi Li, Xiyuan Zhang, Ge’er En, Bo Pang

**Affiliations:** 1 Guang’anmen Hospital, China Academy of Chinese Medical Sciences, Beijing, China; 2 Tianjin University of Traditional Chinese Medicine, Tianjin, China

**Keywords:** NDC1, pan-cancer, pancreatic cancer, immune, prognosis, therapy

## Abstract

NDC1 is a transmembrane nucleoporin that participates in cell mitosis. In the field of oncology, NDC1 has shown its potential as a prognostic marker for multiple tumors. However, pan-cancer analysis of NDC1 to fully explore its role in tumors has not been performed and little is reported on its role in pancreatic cancers. In the present study, a pan-cancer analysis of NDC1 was performed using a bioinformatic approach. Survival analysis was performed by univariate Cox regression analysis and Kaplan-Meier survival analysis. Subsequently, the relationship between NDC1 and immune cell infiltration, TMB/MSI and drug sensitivity was analyzed. Moreover, the mechanism of NDC1 in pancreatic cancer were further analyzed by GSEA, GSVA. Finally, we conducted *in vitro* experiments including MTT, scratch, EdU, and apoptosis assays to explore the function of NDC1 in pancreatic cancer cells. High expression of NDC1 was demonstrated in 28 cancer types. Univariate Cox regression analysis revealed that NDC1 expression was closely associated with the survival outcome of 15 cancer types, and further Kaplan-Meier survival analysis showed negative associations with the progression-free survival in 14 cancers. In addition, a significant association between the NDC1 expression and immune cell infiltration in tumor microenvironment, immune-related genes, common tumor-regulatory and drug sensitivity was observed. Furthermore, NDC1 is abnormally expressed in pancreatic cancer, and is closely related to the prognosis of pancreatic cancer patients and chemosensitivity. The study reveals that NDC1 could be used as a potential immunological, prognostic and therapeutic target for pancreatic cancer.

## INTRODUCTION

Cancer has become the second most critical disease that threatens the health of people, which makes the pathogenesis, development, and treatment of cancer a hot spot in the field of medicine. The Cancer Genome Atlas (TCGA) is a publicly funded project developed by the U.S. National Institutes of Health (NIH) and is a landmark cancer genomics program spanning 33 cancer types [1]. In 2012, the “Pan-Cancer Analysis” project was initiated. The goal of the project is to identify the origin of the tumors, define cancer lineages, and help formulate therapeutic strategies by studying the differences and commonalities between tumors [2, 3].

NDC1 Transmembrane Nucleoporin (NDC1) is a transmembrane nucleoporin, also known as Transmembrane Protein 48 (TMEM48), that contains 656 amino acids involved in 6–7 transmembrane constructs [4, 5]. It participates in cell mitosis by serving as a component of the nuclear pore complex and the spindle. Studies show that NDC1 can control nuclear pore complex (NPC) density and nuclear size in the yeast and early C. elegans embryo [6, 7]. For the past few years, increasing studies have been devoted to the role of NDC1 in tumors. Qiao W et al. [8] found that high expression of NDC1 was associated with higher tumor stage, lymph node metastasis, larger tumor size and shorter survival time in patients with non-small cell lung cancer (NSCLC). He W et al. [9] revealed that NDC1 was a key prognostic factor for esophageal squamous-cell carcinoma. Liu M et al. [10] showed that NDC1 was an independent prognostic factor for colon cancer. Studies have also reported that NDC1 is involved in tumor cell proliferation, migration and invasion. For example, Qiao W et al. [8] reported that suppression of NDC1 expression could lead to apoptosis of NSCLC cells and inhibition of cell adhesion, migration, invasion and tumorigenicity. Akkafa F et al. [11] revealed that miR-421 inhibited NDC1 expression in A549 NSCLC cells, thereby advancing cell apoptosis and increasing the expression of Caspase3, PTEN and TP53. Jiang XY et al. [12] uncovered that NDC1 could advance the initiation and progression of cervical cancer via the Wnt/β-catenin pathway. Qing L et al. [13] showed that NDC1 is a prognostic and immunotherapy predictor of hepatocellular carcinoma and that overexpression of NDC1 can promote the migration and invasion of hepatocellular carcinoma. In the field of oncology, NDC1 has shown its potential as a prognostic marker for multiple tumors. In addition, it is involved in the proliferation, migration and invasion of tumor cells and also affects the chemo-resistance of tumor cells with the activity against apoptosis [14].

The tumor microenvironment (TME) mainly consists of blood vessels, immune cells, fibroblasts, stromal cells, and the extracellular matrix [15]. The interaction between tumor and TME promotes the proliferation, migration and invasion of tumor, and the role of immune cells in TME is particularly important [16]. Although the immune system can eliminate tumors through the cancer immune cycle, tumors appear to ultimately evade immune surveillance by shaping the immunosuppressive microenvironment. Immunotherapy, as a new tumor treatment after surgery, radiotherapy and chemotherapy, can remodel the TME and restore the tumor killing ability of anti-tumor immune cells [17]. Nivolumab, as an inhibitor of programmed death-1, has an objective response rate of 40–45% for non-small cell lung cancer [18]. Therefore, it is particularly important to find tumor immune-related therapeutic targets through TME analysis.

**Figure 1:**
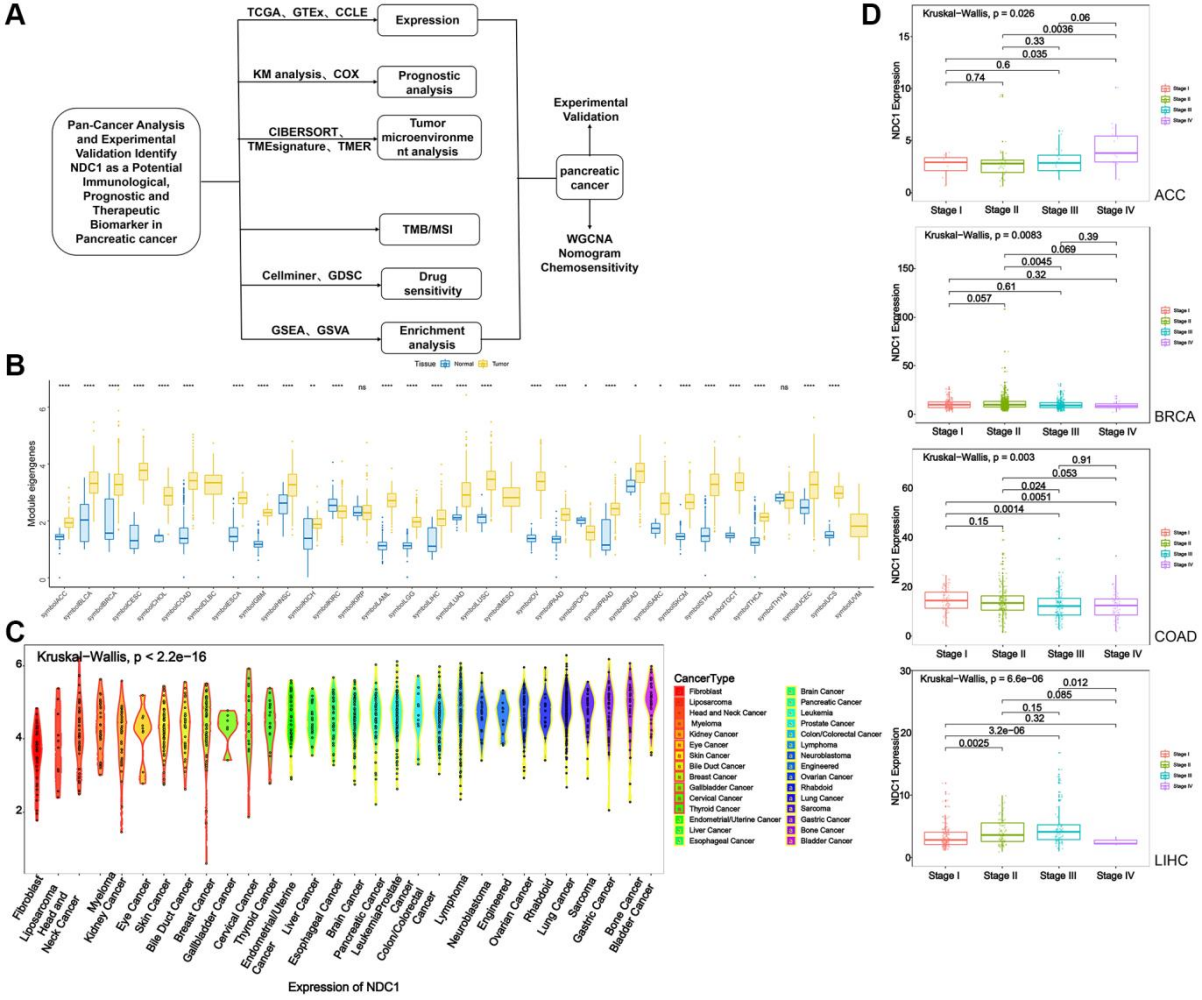
(**A**) Flowchart. (**B**) Expression of NDC1 in pan-cancer based on TCGA and GTEx databases (yellow for tumor tissue and blue for normal tissue). (**C**) Expression of NDC1 in different cell lines from the CCLE database. (**D**) Relationship between NDC1 expression and tumor stage in ACC, BRCA, COAD and LIHC.

In the current study, three databases, TCGA, Genotype-Tissue Expression (GTEx) and Cancer Cell Line Encyclopedia (CCLE), were visited to study the expression of NDC1 in 33 types of cancer and explore its prognostic significance. In the meantime, we also explored the potential association of NDC1 with TME, drug sensitivity, Gene Set Enrichment Analysis (GSEA), Gene Set Variation Analysis (GSVA), tumor mutational burden (TMB), microsatellite instability (MSI). Notably, we focus on pancreatic cancers to analyze the prognostic significance of NDC1. The results revealed that NDC1 is closely related to the immunity in tumor and has the potential as a target for tumor therapy (Figure 1A).

## RESULTS

### Expression and prognosis of NDC1 in pan-cancer

**Figure 2:**
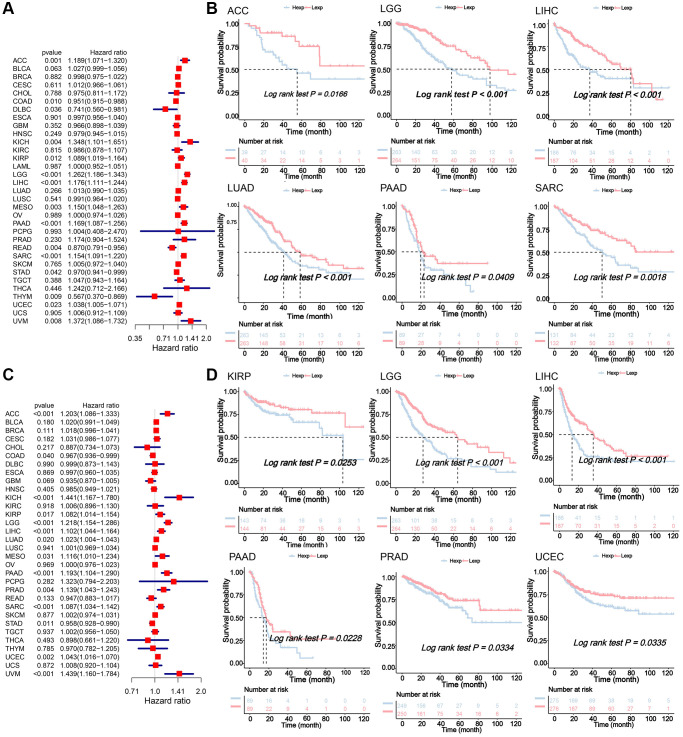
(**A**) Univariate Cox regression analysis for the prognostic significance of NDC1 for OS. HR>1 indicates NDC1 is a risk factor and HR<1 indicates NDC1 is a protective factor. *P* < 0.05 was considered statistically significant. (**B**) Kaplan-Meier survival analysis for OS. *P* < 0.05 was considered statistically significant (Red for high NDC1 expression and blue for low NDC1 expression). (**C**) Univariate Cox regression analysis for the prognostic significance of NDC1 for PFS. HR>1 indicates NDC1 is a risk factor and HR<1 indicates NDC1 is a protective factor. *P* < 0.05 was considered statistically significant. (**D**) Kaplan-Meier survival analysis for PFS. *P* < 0.05 was considered statistically significant (Red for high NDC1 expression and blue for low NDC1 expression).

Expression of NDC1 was analyzed in 33 cancer types based on data from TCGA and GTEx. Higher expression of NDC1 was demonstrated in 26 cancer types, including Adrenocortical carcinoma (ACC), Bladder Urothelial Carcinoma (BLCA), Breast invasive carcinoma (BRCA), Cervical squamous cell carcinoma and endocervical adenocarcinoma (CESC), Cholangiocarcinoma (CHOL), Colon adenocarcinoma (COAD), Esophageal carcinoma (ESCA), Glioblastoma multiforme (GBM), Head and Neck squamous cell carcinoma (HNSC), Kidney Chromophobe (KICH), Acute Myeloid Leukemia (LAML), Brain Lower Grade Glioma (LGG), Liver Hepatocellular carcinoma (LIHC), Lung adenocarcinoma (LUAD), Lung squamous cell carcinoma (LUSC), Ovarian serous cystadenocarcinoma (OV), Pancreatic adenocarcinoma (PAAD), Prostate adenocarcinoma (PRAD), Rectum adenocarcinoma (READ), Sarcoma (SARC), Skin Cutaneous Melanoma (SKCM), Stomach adenocarcinoma (STAD), Testicular Germ Cell Tumors (TGCT), Thyroid carcinoma (THCA), Uterine Corpus Endometrial Carcinoma (UCEC), Uterine Carcinosarcoma (UCS), as compared to the expression in normal tissues (Figure 1B). The expression of NDC1 in different cell lines from the CCLE database was shown in (Figure 1C). In addition, the expression of NDC1 was associated with the tumor stage in ACC, BRCA, COAD and LIHC (Figure 1D). The prognostic significance of NDC1 for overall survival (OS) and progression-free survival (PFS) of cancer patients was analyzed by Univariate Cox regression. It was found that NDC1 expression was closely related to the OS of patients with ACC, COAD, Lymphoid Neoplasm Diffuse Large B-cell Lymphoma (DLBC), KICH, Kidney renal papillary cell carcinoma (KIRP), LGG, LIHC, MESO, PAAD, READ, SARC, STAD, THYM, UCEC and Uveal Melanoma (UVM) (Figure 2A). Notably, high expression of NDC1 was prognostic for poor OS of ACC, LGG, LIHC, LUAD, PAAD and SARC (Figure 2B). In the meantime, NDC1 expression was closely related to the PFS of patients with ACC, COAD, KICH, KIRP, LGG, LIHC, LUAD, Mesothelioma (MESO), PAAD, PRAD, SARC, STAD, UCEC, UVM (Figure 2C), and high expression of NDC1 was prognostic for poor PFS of KIRP, LGG, LIHC, PAAD, PRAD and UCEC (Figure 2D).

### NDC1 expression and immune infiltration

**Figure 3:**
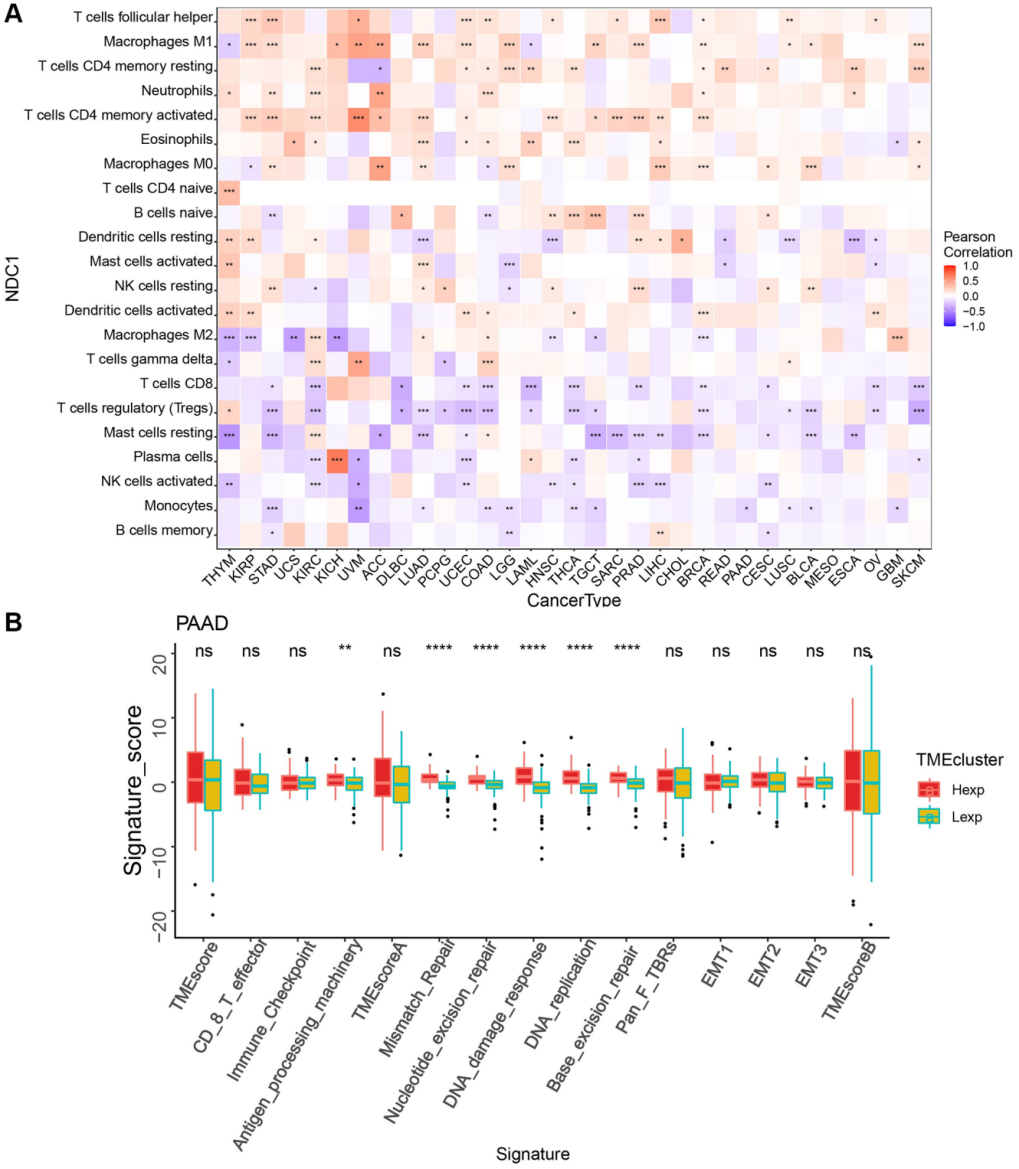
(**A**) Correlation between NDC1 expression and immune cell infiltration (CIBERSORT) (red represents positive correlation, and blue represents negative correlation). (**B**) TME analysis in PAAD (red for high-expression and yellow-green for low-expression).

The TME plays an important role in tumor diagnosis, survival outcome and clinical therapeutic sensitivity. Here, we found that the expression of NDC1 was closely associated with the immune infiltration in TME. Specifically, NDC1 was significantly associated with T cells follicular helper in 11 cancer types, with Macrophages M1 in 16 cancer types and with T cells CD4 memory resting in 12 cancer types (Figure 3A). In addition, analysis in PAAD also showed significant associations with Antigen_processing_machinery, Mismatch_Repair, Nucleotide_excision_repair, DNA_damage_response, DNA_replication, Base_excision_repair (Figure 3B).

### NDC1 expression and immune-related genes/tumor-regulatory genes

**Figure 4:**
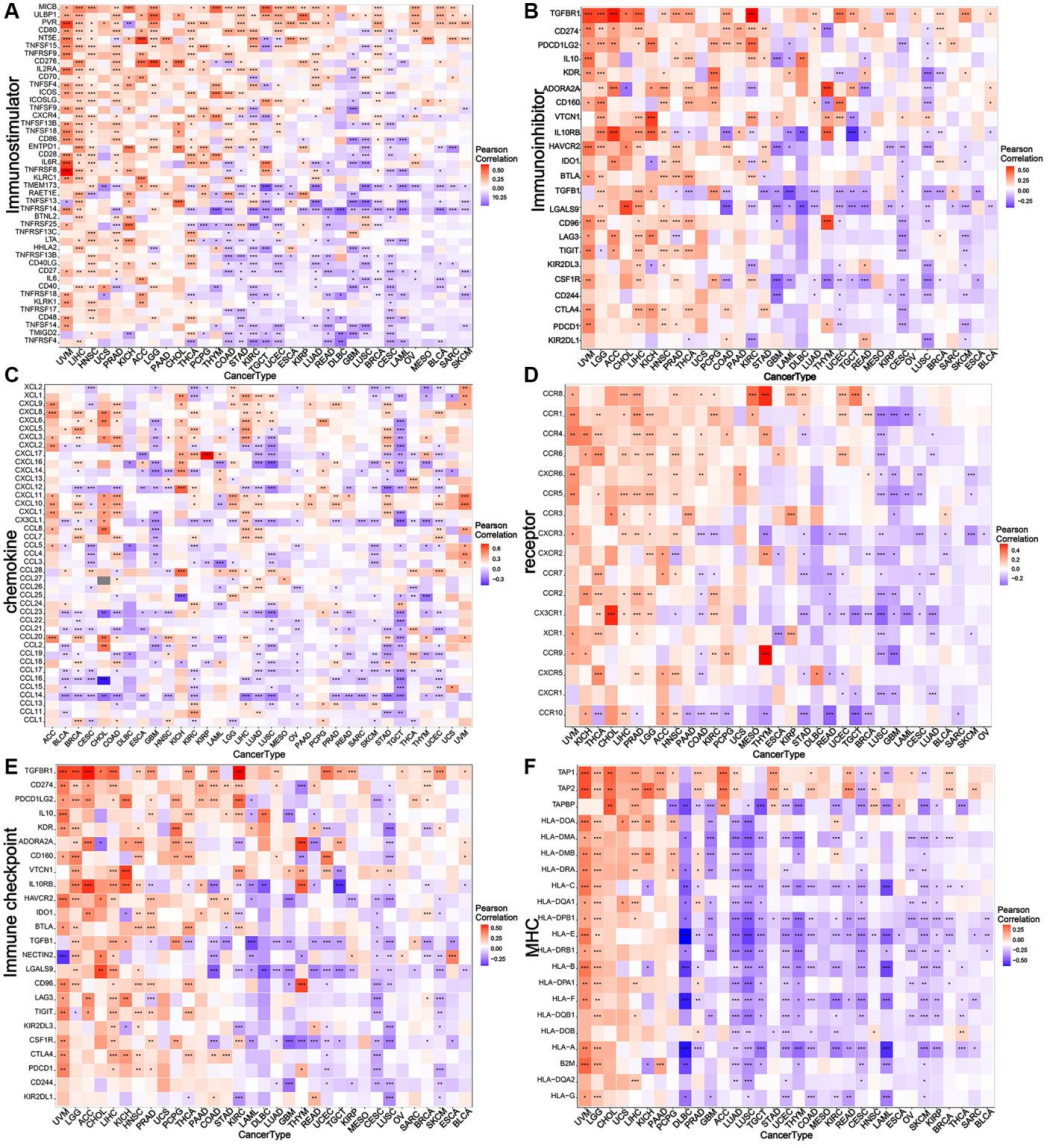
**Relationship between NDC1 expression and immune-related genes.** (**A**–**F**) Associations with MHC. (**A**) immunostimulator, (**B**) immunoinhibitor, (**C**) chemokine, (**D**) receptor, (**E**) immune checkpoint, (**F**) MHC. Red represents positive association and blue represents negative association.

**Figure 5:**
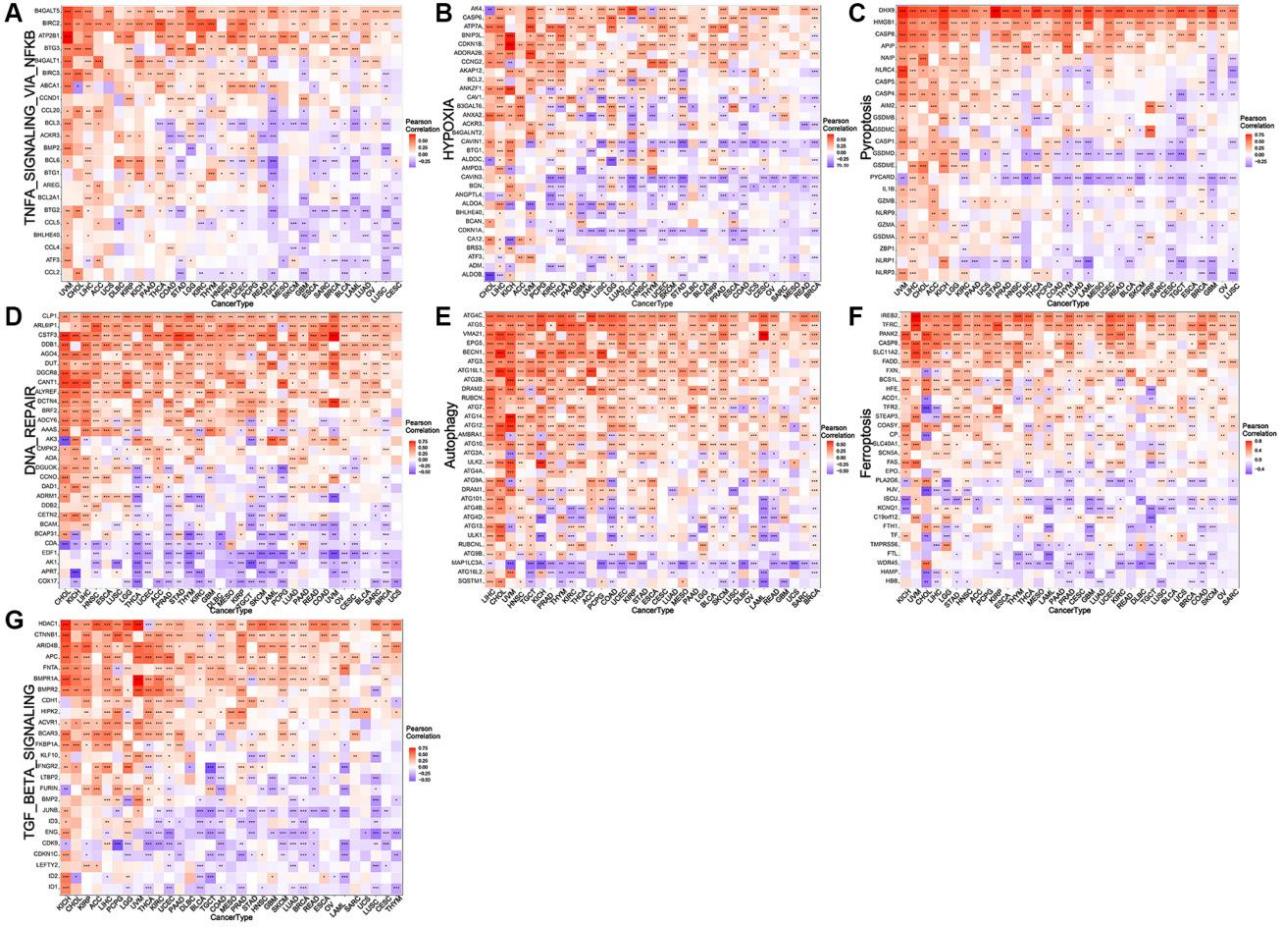
**Relationship between NDC1 expression and common tumor-regulatory genes.** (**A**–**G**) Associations with genes involved in (**A**) TNFA SIGNALING VIA NFKB, (**B**) hypoxia, (**C**) pyroptosis, (**D**) DNA repair, (**E**) autophagy, (**F**) and ferroptosis. (**G**) TGF BETA SIGNALING. Red represents positive association and blue represents negative association.

Correlation analysis was performed to analyze the associations of NDC1 expression in pan-cancer with multiple immune-related genes, including major histocompatibility complexes (MHC), immunostimulator, immunoinhibitor, chemokine and chemokine receptor, immune checkpoint. The results revealed that the NDC1 expression was significantly associated with almost all the immune-related genes (Figure 4). Additionally, NDC1 was also demonstrated to be associated with the common tumor-regulatory genes involved in TGF BETA SIGNALING, TNFA SIGNALING, hypoxia, pyroptosis, DNA repair, autophagy and ferroptosis (Figure 5).

### NDC1 expression and TMB/MSI in pan-cancer

**Figure 6:**
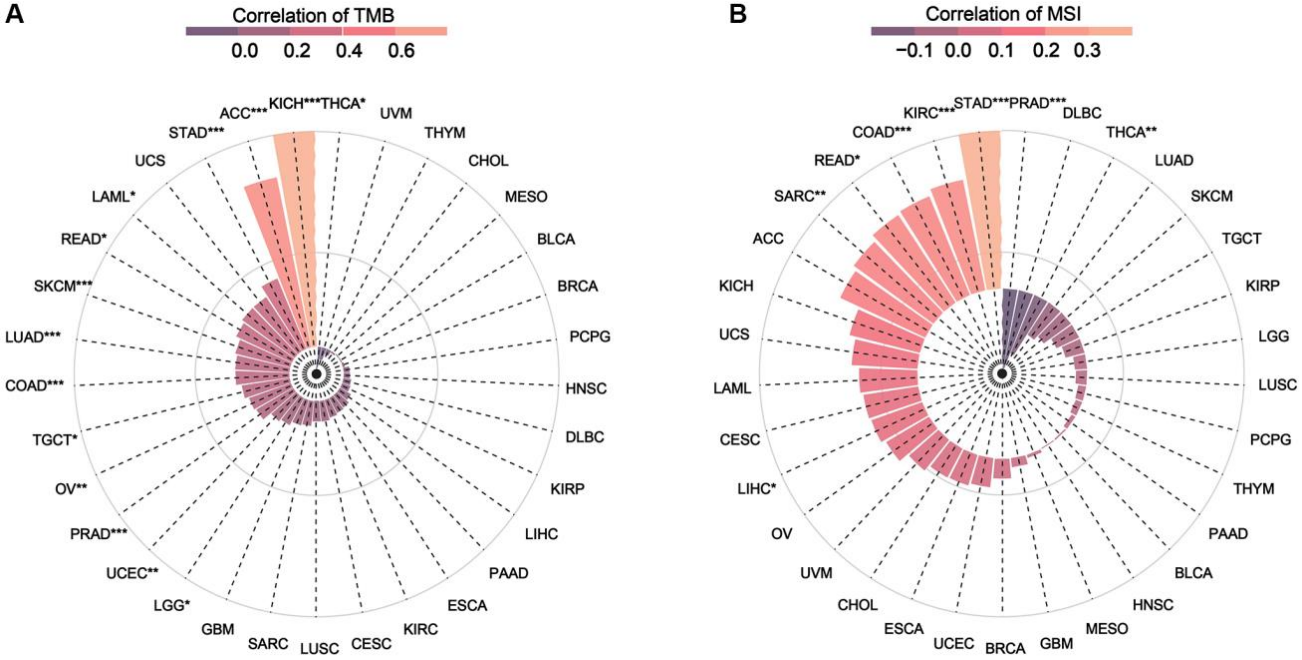
(**A**) Relationship between TMB and NDC1 expression in pan-cancer. (**B**) Relationship between MSI and NDC1 expression in pan-cancer. The correlation increases from purple to pink.

TMB and MSI are two novel biomarkers for tumor immunotherapy. TMB refers to the number of somatic non-synonymous mutations in a specific genomic region, which can indirectly reflect the ability and degree of neoantigen production of tumors and predict the efficacy of immunotherapy in a variety of tumors [18–20]. MSI refers to the phenomenon of short, repetitive DNA sequence length changes caused by insertion or deletion mutations during DNA replication, and is often caused by defective MMR function [21]. Correlation analysis revealed that NDC1 expression was significantly associated with TMB in THCA, KICH, ACC, STAD, LAML, READ, SKCM, LUAD, COAD, TGCT, OV, PRAD, UCEC, LGG (Figure 6A) and evidently associated with MSI in PRAD, THCA, STAD, KIRC, COAD, READ, SARC, LIHC (Figure 6B).

### NDC1 expression and drug sensitivity in pan-cancer

**Figure 7:**
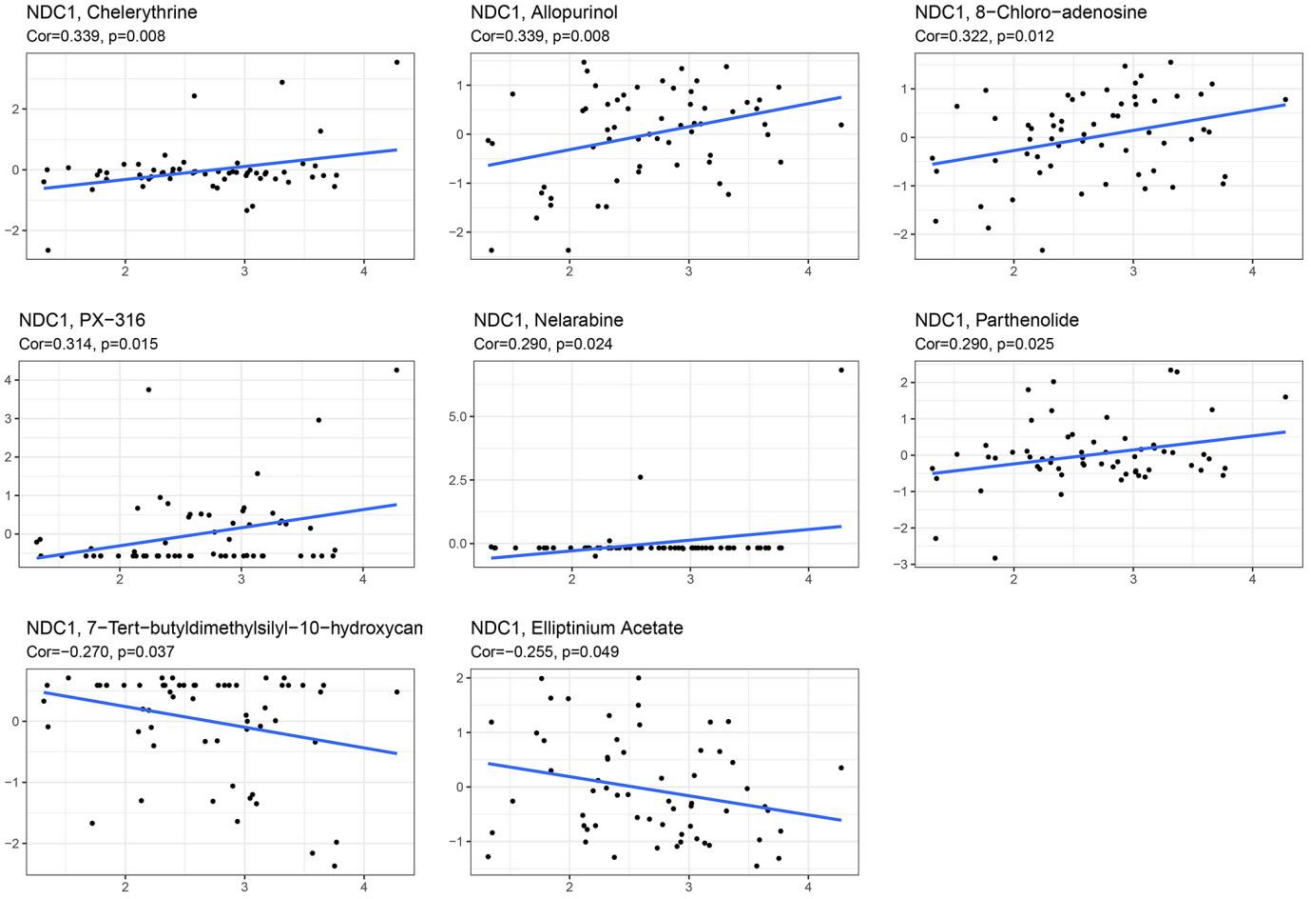
Correlation between NDC1 expression and IC50 of multiple anti-tumor drugs, including Chelerythrine, Allopurinol, 8-Chloro-adenosine, PX-316, Nelarabine, Parthenolide, 7-Tert-butyldimethylsilyl-10-hydroxycamptothecin and Elliptinium Acetate.

The combination of surgery with chemotherapy has significant therapeutic effect in treatment of early tumors, for example, patients with pancreatic cancer who received adjuvant chemotherapy with gemcitabine after surgery had a higher median overall survival than those who received no chemotherapy compared with those who received observation, and dual therapy provided a higher survival benefit than gemcitabine alone [22]. Here, the CellMiner database was visited to analyze the relationship between NDC1 expression and some common anti-tumor drugs. It was found that high NDC1 expression was highly associated with the development of resistance to multiple anti-tumor drugs (Table 1). More specifically, NDC1 expression was positively associated with the drug resistance of Chelerythrine, Allopurinol, 8-Chloro-adenosine, PX-316, Nelarabine, Parthenolide, but negatively associated with 7-Tert-butyldimethylsilyl-10-hydroxycamptothecin and Elliptinium Acetate (Figure 7).

**Table 1:** Correlation between NDC1 expression and IC50 of multiple anti-tumor drugs.

**Drug**	**cor**	***p* value**
Chelerythrine	0.339293583	0.008000628
Allopurinol	0.339167212	0.008025561
8-Chloro-adenosine	0.322285759	0.012028721
PX-316	0.314078877	0.014531203
Nelarabine	0.290349649	0.024420463
Parthenolide	0.289989421	0.024606158
7-Tert-butyldimethylsilyl-10-hydroxycamptothecin	−0.270147786	0.036840909
Elliptinium Acetate	−0.255284596	0.049001315

### GSVA and GSEA of NDC1 in PAAD

**Figure 8:**
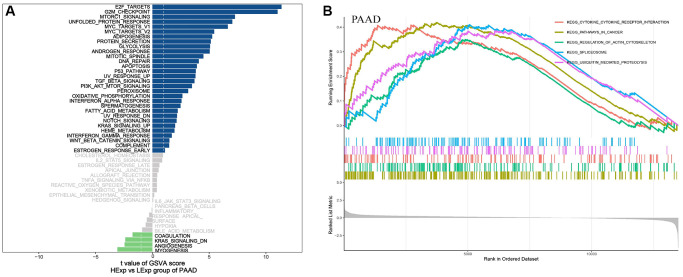
(**A**) GSVA pathway enrichment in PAAD. Blue represents the enriched pathways in high NDC1 expression group and green represents the enriched pathways in low NDC1 expression group. (**B**) GSEA pathway enrichment in PAAD. The left shows the enrichment score and the right shows the main enriched pathways.

To further study the molecular mechanisms of NDC1 in PAAD, PAAD samples were divided into high- and low-expression groups according to the median NDC1 expression. GSVA was performed and the results showed that high NDC1 was mainly involved in E2F_TARGETS, G2M_CHECKPOINT, MTORC1_SIGNALING, UNFOLDED_PROTEIN_RESPONSE and MYC_TARGETS_V1 in PAAD (Figure 8A). GSEA was performed and the results showed that high NDC1 was mainly involved in CYTOKINE_CYTOKINE_RECEPTOR_INTERACTION, PATHWAYS_IN_CANCER, REGULATION_OF_ACTIN_CYTOSKELETON, SPLICEOSOME, UBIQUITIN_MEDIATED_PROTEOLYSIS in PAAD (Figure 8B).

### Weighted gene coexpression network analysis (WGCNA) in PAAD

**Figure 9:**
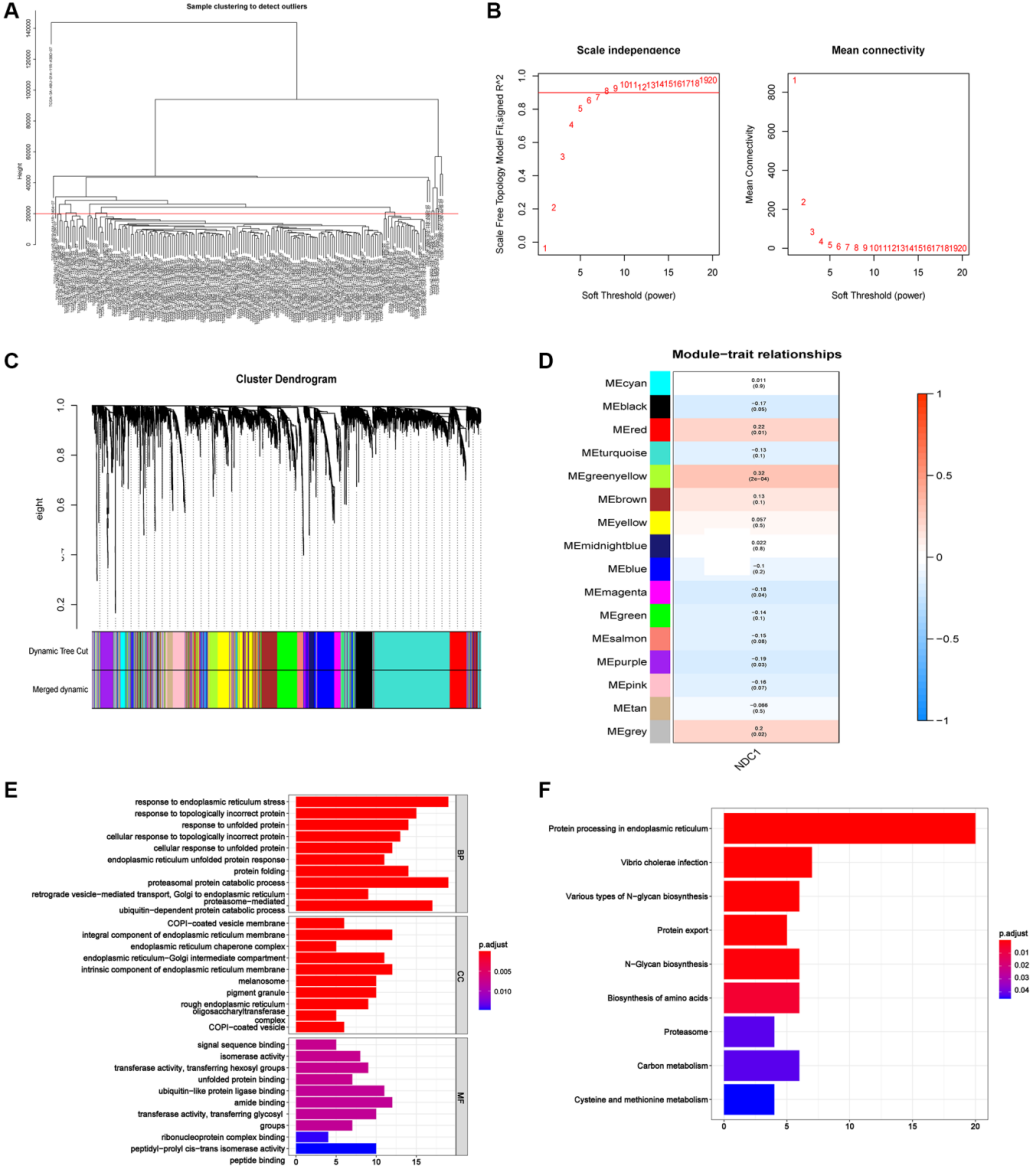
(**A**) The sample clustering dendrogram. (**B**) Determination of soft-threshold power. (**C**) Dendrogram of all differentially expressed genes clustered based on the measurement of dissimilarity. The color band shows the results obtained from the automatic single-block analysis. (**D**) Module-trait correlation in PAAD. The upper number is the correlation coefficient and the lower number in bracket is the *p* value. (**E**) The most enriched GO terms of the genes in MEgreenyellow module. (**F**) The most enriched KEGG pathways of the genes in MEgreenyellow module.

We further constructed WGCNA network based on PAAD expression profile data to explore NDC1-related co-expression network in PAAD. The outlier samples were deleted, and the samples with a height below 20000 were retained for subsequent analysis after clustering the samples. The β was set as 6 according to “sft$powerEstimate” function. A total of 16 gene modules were obtained, including MEblack (270), MEblue (504), MEbrown (491), MEcyan (75), MEgreen (365), MEgreenyellow (166), MEgrey (278), MEmagenta (213), MEmidnightblue (68), MEpink (225), MEpurple (179), MEred (319), MEsalmon (85), MEtan (98), MEturquoise (1,280), and MEyellow (384). Module-trait correlation analysis found that the MEgreenyellow module was the highest associated with the clinical traits of PAAD (cor = 0.32, *p* = (2e-04)) (Figure 9A–9D). Genes of the MEgreenyellow module was further analyzed by Gene Ontology (GO) and Kyoto Encyclopedia of Genes and Genomes (KEGG) enrichment analysis. The most enriched GO terms were response to endoplasmic reticulum stress, response to topologically incorrect protein, response to unfolded protein (Figure 9E), and the most enriched KEGG pathways were involved in Protein processing in endoplasmic reticulum, Vibrio cholerae infection, Various types of N−glycan biosynthesis (Figure 9F).

### Prognostic significance of NDC1 in PAAD

**Figure 10:**
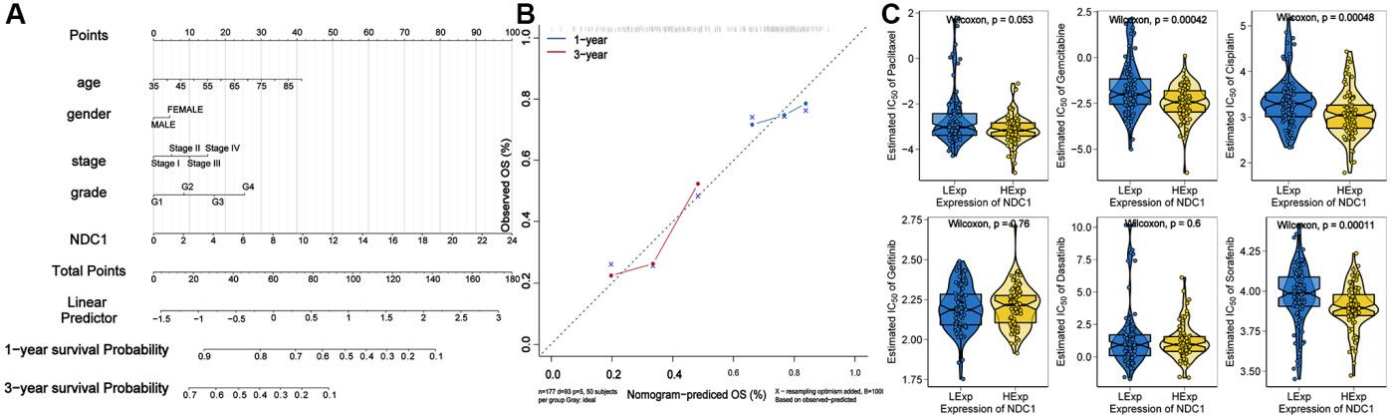
(**A**) Nomogram established for prognosis of PAAD. (**B**) Calibration curves for 1- and 3-year survival of PAAD. (**C**) Chemosensitivity of NDC1 in PAAD.

A nomogram for prognosis of PAAD based on NDC1 expression and several significant clinical characteristics was established. NDC1 expression was found to be significantly prognostic for survival of PAAD (Figure 10A). In addition, this study draws correction curves for one year and three years at the same time, and finds that the OS predicted by Nomo chart is in good agreement with the actual observed OS, and is close to the slope, suggesting that Nomo chart has good prediction efficiency. (Figure 10B).

### Chemosensitivity of NDC1 in PAAD

Based on the drug sensitivity data of the GDSC database, our study used the R package “pRRophetic” to predict the chemosensitivity of each tumor sample, and further explores the sensitivity of NDC1 to common chemotherapeutic drugs in PAAD. The results showed that the expression of NDC1 in PAAD samples was correlated with the sensitivity of patients to Gemcitabine, Cisplatin, and Sorafenib (Figure 10C).

### Knockdown of NDC1 inhibits the proliferation, migration and apoptosis in PC

**Figure 11:**
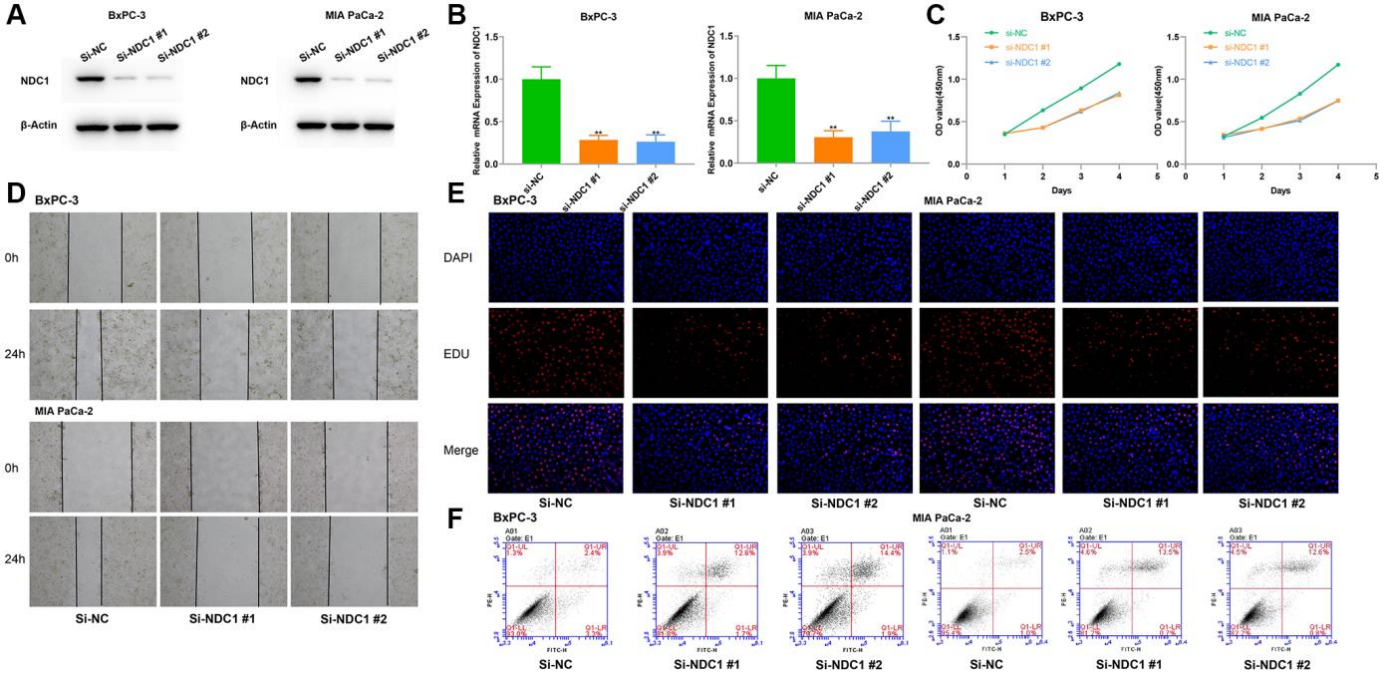
**Cellular functions of NDC1 in pancreatic cancer.** (**A**) Western blot and (**B**) Real-time quantitative PCR detection after siRNA mediated knockdown of NDC1 in BxPC-3 and MIA PaCa-2 cells. (**C**) MTT assays. (**D**) Scratch assays. (**E**) EdU assays. (**F**) Cell apoptosis assays. ^*^P < 0.05.

The effect of NDC1 on pancreatic cancer cell function was determined by loss-of-function experiments expressed in pancreatic cancer (PC) and contributes to drug resistance and poor prognosis. *In vitro*, we silenced the expression of NDC1 in human pancreatic cancer cell lines BxPC-3 and MIA PaCa-2, respectively, and the interference efficiency was verified by RT-qPCR and Western blot analysis (Figure 11A, 11B). The results of Our pan-cancer analysis showed that NDC1 is highly MTT and 5-ethynyl-20 deoxyuridine (EdU) experiments showed that silencing NDC1 could significantly inhibit the proliferation of pancreatic cancer cells (Figure 11C, 11E). Scratch assay and apoptosis assay showed that downregulation of NDC1 expression reduced cell migration ability and promoted cell apoptosis (Figure 11D, 11F).

## DISCUSSION

In this study, the expression level of NDC1 in 33 tumors was evaluated by pan-cancer analysis, and the effects of NDC1 on the proliferation, invasion and migration of pancreatic cancer were experimentally verified for the first time. In the meantime, we also found that NDC1 expression was closely associated with the OS and PFS of multiple cancer types. The most important finding of the study is the significant association with tumor immune infiltration. It is noting that NDC1 expression was profoundly associated with almost all immune-related genes and common tumor-regulatory genes. GSEA and GSVA were performed to analyze the NDC1-mediated signaling pathways. Finally, we analyzed the prognostic significance and chemosensitivity of NDC1 in PAAD, and explored possible molecular mechanisms using WGCNA, GO annotation and KEGG pathway enrichment analysis.

At present, studies on NDC1 mainly are involved in lung cancer, colon cancer, cervical cancer, esophageal cancer and hepatocellular carcinoma [8–13], and there was no previous report in pancreatic cancers. The latest research revealed that pancreatic cancer is a highly malignant cancer and the 5-year survival rate is reported to 3% only [23]. Given the not obvious clinical symptoms in early stages, pancreatic cancer is readily to be diagnosed at an advanced stage, missing the optimal timing of treatment. Therefore, it is of crucial significance to conduct studies on early diagnosis, prognosis and treatment of the pancreatic cancer. In order to improve the early diagnosis rate and clinical efficacy of pancreatic cancer, some scholars have discovered and verified some biomarkers of the pancreatic cancer in recent years, such as S100 Calcium Binding Protein A6 (S100A6) [24] and Glypican 2 (GPC2) [25]. In the current study, we performed WGCNA, GO annotation and KEGG pathway enrichment analysis to obtain co-expressed genes of NDC1 in PAAD and subsequently explored possible mechanisms. Moreover, corresponding nomograms were established and proved that NDC1 was significantly prognostic for survival of PAAD.

Immunotherapy has emerged as an effective treatment for cancer due to the fact that, tumor cells can escape from the attacks by the body’s immune system, which allows them to proliferate with no restrictions [26]. This therapy works with the active and passive tumor targeting strategies [27], via a variety of approaches, including recombinant vaccine, ICI, adoptive cell transfer (ACT), cytokine therapy and oncolytic virus therapy [28, 29]. In clinical setting, immunotherapy has shown satisfactory effect when combined with the traditional chemotherapy and radiotherapy [30, 31]. Immune cells are important in immunotherapy, and a good understanding on the immune infiltration in TME is key to the improvement of therapeutic outcome and development of new immunotherapeutic strategies [32]. In the present study, CIBERSORT algorithm was applied. The expression of NDC1 is generally associated with the high expression of T cells follicular helper, Neutrophils, T cells CD4 memory activated and the low expression of T cells CD8, NK cells activated and Monocytes in 33 tumor types. This suggests that we should focus on these immune cells in the immunotherapy study of NDC1. In PAAD, NDC1 expression was analyzed to show a significant association with the infiltration of T cells regulatory. These results, to some extent, provide evidence for the development of immunotherapy for pancreatic cancer in clinics.

TMB and MSI are recognized biomarkers of response to immune checkpoint inhibitors (ICIs) [33]. The two are independent of each other and also correlated with each other. Despite multiple tumors with high MSI having high TMB as well, there are also some tumors with high TMB do not show MSI deficiency [34]. It is well believed that tumorigenesis is accompanied by gene mutations [35]. Proteins expressed by the mutated genes can be recognized by MHC, which then triggers a series of responses reacted by the body’s immune system [36–38]. The more mutations, the more antigens and the higher possibility to be recognized by the immune system. Both TMB and MSI can be used to recognize mutated genes. In this context, patients with high TMB and MSI can response better to immunotherapy [39, 40]. Combining the correlation between NDC1 expression and some common tumor-regulatory genes and the enriched pathways identified by GSEA and GSVA, we speculated that NDC1 might be implicated in tumor via other pathways, except immune-related pathways, which requires further exploration.

At present, the treatment of tumor includes radiotherapy, chemotherapy, surgery, immunotherapy, etc., but chemotherapy is still the most common treatment. Clinically, patients with long-term chemotherapy are prone to multidrug resistance, resulting in a decline in the treatment effect. Studies have shown that over 90% mortality of cancer patients is attributed to drug resistance [41]. Modified leucovorin, 5-fluorouracil, irinotecan and oxaliplatin (mFOLFIRINOX) are the first-line chemotherapy regimens for pancreatic cancer [42]. Compared with finding new chemotherapeutic drugs, it is more important to solve the problem of chemotherapeutic drug resistance. In this study, it is proposed for the first time that NDC1 is related to tumor chemotherapy resistance, and then we need to analyze the specific drugs targeting NDC1 drug sensitivity to pancreatic cancer through in-depth study, in order to further guide clinical drug use.

## CONCLUSION

To conclude, we identified the importance of NDC1, particularly in pancreatic cancer, by pan-cancer analysis. This study proved that NDC1 could be used as a potential immunological, prognostic and therapeutic target for pancreatic cancer. However, this study has certain limitations, including the lack of *in vivo* studies on NDC1 in pancreatic cancer and the specific molecular biological mechanism of NDC1 regulation of pancreatic cancer, etc. Further studies are still needed to guide clinical diagnosis and treatment in the future.

## METHODS

### Data acquisition and differential analysis

TCGA (https://portal.gdc.cancer.gov/) is currently known as the largest database that covers cancer genomics information including gene expression data, copy number variation (CNV) and single nucleotide polymorphism (SNP), etc. Here, mRNA expression data and SNP data of 33 cancer types were downloaded from the TCGA database and performed log2 normalization of gene expression levels for subsequent analysis. Gene expression data of different tissue samples were also obtained from the GTEx database (https://commonfund.nih.gov/GTEx). Data from the TCGA and GTEx databases were combined and corrected through the normalize Between Arrays function, after which differential analysis was performed to analyze the differential expression of NDC1 in different cancers. In addition, CCLE database (https://portals.broadinstitute.org/ccle/) was visited to download tumor cell data and then the expression of NDC1 in corresponding tumor tissues was analyzed. The relationship between the NDC1 expression and tumor stage was also explored. According to the expression level of NDC1 gene, the expression difference of NDC1 expression in different stages of tumor was analyzed. The comparison between two groups was conducted by Wilcox test, and the comparison between multiple groups was conducted by Kruskal test.

### Prognostic analysis

Survival data of TCGA samples, including OS and PFS, were downloaded from the Xena database (https://xenabrowser.net/) to study the prognostic significance of NDC1. Kaplan-Meier method was applied for survival analysis with packages “survival” and “survminer”. Univariate Cox regression analysis was conducted to discuss the prognostic significance of NDC1 using packages “survival” and “forestplot” in pan-cancer.

### Immune cell infiltration analysis

CIBERSORT algorithm was adopted to analyze the RNA-seq data of 33 cancer types. The infiltration abundance of immune cells was inferred and the association with NDC1 expression was analyzed. In the meantime, the TISIDB website (http://cis.hku.hk/TISIDB) was visited to explore the potential associations of NDC1 expression with immune-related genes, such as chemokine, immunoinhibitor, immunostimulator and MHC.

### TMB and MSI analysis

TMB is defined as the number of somatic, coding, base substitution, and indel mutations per megabase of genome examined. Here, TMB was defined as the ratio of the length of protein-coding region of non-synonymous variants to the total length of protein-coding region, based on the variation frequency and number/exon length of each tumor sample. The MSI of each TCGA sample was derived from a previous published literature [43].

### Drug sensitivity analysis

The CellMiner database (https://discover.nci.nih.gov/cellminer/home.do) is designed for the cancer research community to facilitate integration and study of molecular and pharmacological data for the NCI-60 cancerous cell lines, which are a panel of 60 diverse human cancer cell lines used by the Developmental Therapeutics Program of the U.S. NCI to screen over 100,000 chemical compounds and natural products. The NCI-60 panel is now widely used in anti-cancer drug tests. NCI-60 dataset of drug sensitivity and RNA-seq data were obtained to study the relationship between NDC1 expression and anti-tumor drug sensitivity.

### GSVA

GSVA is a nonparametric, unsupervised method used to perform transcriptome and gene set enrichment analysis. It can confer an enrichment score to a certain gene set of each sample and estimate variation of pathway activity by transforming gene expression variations. Here, related gene sets were obtained from Molecular signatures database (v7.0) to estimate the variation of pathway activity in different samples.

### GSEA

GSEA ranks genes based on their differential expression between two sample types and then uses a pre-defined gene set to study whether the gene set is enriched in the top or the bottom of the rank list. GSEA was performed using packages “ClusterProfiler” and “enrichplot” to analyze the differential signaling pathways between the high- and low-expression groups stratified by the median NDC1 expression, and possible molecular mechanisms were discussed.

### Weighted correlation network analysis (WGCNA)

WGCNA was performed to find co-expressed genes of NDC1 and study the association with clinical traits. The R package “WGCNA” was used to establish a co-expression network based on the top 5,000 genes with differential expression, the “hclust” function was used to cluster samples, and the “pickSoftThreshold” function was used to calculate the soft threshold. The soft-thresholding power (β) was set as 6 for PAAD. The weighted adjacency matrix was transformed to a topological overlap matrix (TOM) to estimate network connectivity, and a clustering tree was generated using the hierarchical clustering method. Each branch represents a gene module and differs by colors. According to the weighted correlation coefficient of each gene, genes of similar expression patterns were clustered into the same module.

### Enrichment analysis

The key gene module was identified. R package “ClusterProfiler” was used to perform functional annotation for the genes in the key module. GO and KEGG were employed to estimate related biological functions. *P* < 0.05 or *q* < 0.05 was considered statistically significant.

### Nomogram establishment

Nomogram is generally established for clinical use. It provides a scale according to the contribution (multi-variate regression coefficient) of each variable in prognosis (gene expression and several significant clinical features here). Each variable was conferred a score and the total score was used to estimate the prognosis of patients. Calibration curve is a tool used to evaluate the accuracy of model prediction results. cph function of RMS software package is used to construct Cox proportional risk regression model according to clinical symptoms and NDC1 expression, and the calibration curve of the model is drawn.

### Chemosensitivity

Based on the largest pharmacogenomics database (GDSC Cancer Drug Sensitivity Genomics Database, https://www.cancerrxgene.org/), we used the R package “pRRophetic” to predict the chemosensitivity, chemotherapeutic drugs of each tumor sample Includes Paclitaxel, Gemcitabine, Cisplatin, Gefitinib, Dasatinib and Sorafenib.

### Cell culture and siRNAs transfection

The human pancreatic cancer cell lines BxPC-3 and MIA-PaCa2 were procured from the China Center for Type Culture Collection (CCTCC). The cells were grown in Dulbecco’s Modified Eagle Medium (DMEM; HyClone, Cat#SH30022.01, USA) media with 10% Certified Foetal Bovine Serum (FBS; BI, Cat#04-001-1ACS, Israel) and 1% penicillin/streptomycin (Invitrogen, Grand Island, NY, USA). The cells were cultured at 37°C in a 5% CO_2_ incubator. The BxPC-3 and MIA-PaCa2 cell lines were transfected by siRNAs designed and synthesized by RiboBio Co., Ltd., (Guangzhou, China) using Opti-MEM (Invitrogen, Cat#31985-070, USA) medium and Lipofectamine™ 3000 Transfection Reagent (Invitrogen, Cat#L3000-008, USA). The cell lines were randomly divided into two groups: the si-NC group transfected with the scrambled siRNA was considered as the negative control, the si-NDC1 group was transfected with the siRNA specific for NDC1.

### Western blot analysis

After the cells were transfected for 48 h, Western blot analysis was performed as previously described [44]. β-actin was used as normalization. All the blots were incubated with the respective primary antibodies, anti-NDC1 (GeneTex, CTX120091, China), anti-E-cadherin (Proteintech Group, Cat#20874-1-AP, China), anti-N-cadherin (Proteintech Group, Cat#22018-1-AP, China), anti-β-actin (Proteintech Group, Cat #81115-1-RR, China). The protein bands were visualized with electrochemiluminescence (Bio-Rad, USA).

### qRT-PCR

According to the manufacturer’s protocol, ChamQ SYBR qPCR Master Mix (Vazyme, Nanjing, China, Cat#Q311-02) on an RT-PCR system (CFX96 Touch; Bio-Rad, USA) are used to verify the interference efficiency of transfection of siRNAs. The primers sequences used for qRT-PCR were obtained from Applied Biosystems (Ribo, Guangzhou, China) as below (5′–3′): CATACTGTGGCGCGTTTTGG (forward primer), GCAGGGCTACCAAAGCTGTTA (reverse primer). Relative gene expression levels were calculated according to the 2−ΔΔCt value method.

### Cell proliferation, migration and apoptosis assays

The effect of NDC1 on the proliferation of BxPC-3 and MIA-PaCa2 cells was assayed using MTT and EdU labelling assays according to the manufacturers’ instructions. The transfected cells were inoculated into 96-well plates with 5 × 10^3^ cells per well. At 24, 48, 72, 96 hours, 20 μL MTT solution was added to the medium and cells were incubated for 4 hours. After discarding the medium, 200 μL DMSO was added to the cells and the formazan crystal was dissolved for 15 minutes. The optical density (OD) value at 450 nm was detected by the enzyme-linked immunometric meter (Thermo Fisher Scientific). For the EdU labeling assay, we used an EdU Cell Proliferation Kit with Alexa Fluor 555 (Beyotime, Cat#C0075L, China) to determine cell viability according to the manufacturer’s instructions.

BxPC-3 and MIA-PaCa2 cells were seeded in each well of a 6-well plate to form a monolayer overnight. A 200 μL pipette tip was used to create an artificial wound. After washing with phosphate-buffered saline (PBS) twice, cells were cultured in DMEM with 2% FBS for 24 h in a 37°C incubator, and wounds were visualized at 0 and 24 h. The distance of the wounds was measured using Adobe Illustrator CC 2018 software.

The apoptotic cells were detected using annexin V-FITC along with the PI solution, by flow cytometry assay according to the manufacturer’s instruction (Annexin V-FITC apoptosis detection kit, Vazyme, Cat# A211-02, China). The experiments were performed three times in triplicate.

### Statistical analysis

R 4.0 was applied to complete all statistical analyses. Univariate Cox regression analysis was performed, and Hazards ratios (HR) along with 95% confidence interval (CI) were calculated. Kaplan-Meier curve was generated to study the survival of patients with high- and low-expression of NDC1. GraphPad Prism 9.5 was used to analyze the data. Two-sided *P* < 0.05 was considered statistically significant.

### Data availability statement

The data used to support the findings of this study are available from the corresponding authors upon request.
